# Physical Activity and Perceived Support among Adolescents According to Sex and Municipality

**DOI:** 10.3390/healthcare12151512

**Published:** 2024-07-30

**Authors:** Daniel Sanz-Martín, Germán Ruiz-Tendero, José Manuel Alonso-Vargas, Eduardo Melguizo-Ibáñez

**Affiliations:** 1 Faculty of Humanities and Social Sciences, Universidad Isabel I, 09003 Burgos, Spain; 2 Department of Languages, Arts and Physical Education Teaching, Faculty of Education, Complutense University of Madrid, 28040 Madrid, Spain; german.ruiz@edu.ucm.es; 3 Department of Didactics Musical, Plastic and Corporal Expression, Faculty of Education Science, University of Granada, 18071 Granada, Spain; josemalonsov@correo.ugr.es (J.M.A.-V.); emelguizo@ugr.es (E.M.-I.)

**Keywords:** intensity of physical activity, family, friends, rural area, urban area

## Abstract

A cross-sectional study was conducted with three aims: (1) to determine the degree of compliance with physical activity recommendations among adolescents according to sex and place of residence, (2) to determine the perceived support of family and friends for physical activity among adolescents according to sex and place of residence, and (3) to analyse the influence of family and friends support on compliance with physical activity recommendations among adolescents according to sex and place of residence. A total of 694 adolescents from an inland area of Spain (14.06 ± 1.27 years) participated. Four one-day physical activity questionnaires were administered to assess physical activity and the Parental Support Scale and Peer Support to measure perceived support from family and friends regarding physical activity. Chi-square, Crammer’s V and Student’s tests were calculated to identify differences between variables according to sex and municipality of residence. Moreover, the initial binary logistic regression model was constructed to predict the physical activity compliance. Differences in adherence to physical activity recommendations were found according to the sex of the students (rural area: x^2^ = 4.192, *p* < 0.05; V = 0.106, *p* < 0.05; urban area: x^2^ = 8.999, *p* < 0.05; V = 0.167, *p* < 0.01). There were also sex differences in rural areas on items related to families providing transport (t = 3.878, *p* ≤ 0.001; d = 0.40) and doing physical activity together (t = 4.974, *p* ≤ 0.001; d = 0.50). It is concluded that most adolescents do not comply with physical activity recommendations. In addition, 30.4% of urban girls’ compliance was predicted by the perception that their family members saw them doing physical activity and doing it with friends.

## 1. Introduction

Physical activity is currently defined as any physical movement performed by muscular contraction that results in energy expenditure greater than basal metabolism [1]. Regular physical activity has been scientifically proven to have psychological, physiological and social benefits [2]. Similarly, the World Health Organization [2] states that young people aged 5–17 years should engage in 60 min of moderate-to-vigorous physical activity per day for health benefits.

The physical activity levels of different population groups are influenced by different variables [3]. Within the adolescent population, sex and age have been identified as the most influential variables in engaging in physical activity [4,5]. Knowledge of the factors that condition the practice of physical activity outside school hours facilitates the implementation of interventions to increase levels of physical activity [6].

Although there is no single trend in the findings of different studies, another factor that seems to influence young people’s physical activity levels is social support [7]. Social support can be defined as the help and assistance exchanged through various interpersonal relationships [8]. There are eight types of social support, which fall into three categories: emotional, informational and material [9]. In the adolescent population, it has been observed that there are differences in physical activity as a function of social support [10]. Similarly, it has been found that males’ physical activity practices are oriented towards peer social support, whereas females’ physical activity practices are oriented towards family support [11].

Another factor influencing physical activity levels is the municipality in which young people live [12]. According to the Organization for Economic Cooperation and Development [13], a rural municipality is one with a population density of less than 150 inhabitants per km^2^ and an urban municipality is one with a population density of more than 150 inhabitants per km^2^. In recent years, Spain’s rural areas have been characterized by an increase in industrial agriculture, a decrease in population and the proliferation of technological media [14]. Studies have focused on comparing physical activity levels according to where young people live [11,12]. These have shown that young people living in urban areas have higher energy expenditure and more time spent in physical activity than those living in rural areas [11,12].

In terms of the influence of physical activity factors, ecological frameworks consider that multiple systems influence the development of people’s well-being across the lifespan [15]. For example, the Bronfenbrenner model considers family and friends as part of the microsystem and the gender of the individual [16].

Numerous studies have shown that more than 71% of adolescents do not meet the daily recommendations for moderate-to-vigorous physical activity [4,17]. Other studies have also analysed the influence of adolescents’ family and friends on their physical activity levels [11,18]. Differences in physical activity levels between rural and urban adolescents have also been investigated [19,20]. Despite the existence of such research on physical activity factors, there are few existing studies comparing the relationship between physical activity and social support between rural and urban young people [11], and further research is needed to better understand the mechanisms by which these factors influence physical activity [21,22]. Similarly, no previous research has been identified that analyses the influence of support from family and friends on physical activity among urban and rural youth in economically disadvantaged and sparsely populated areas such as Soria (Spain), so this study may open a future line of research in the existing scientific literature.

Soria is a province of the autonomous community of Castilla y León and is the penultimate province in Spain with the lowest GDP [23]. The province is divided into 183 municipalities, of which 170 have less than 1000 inhabitants [23] and only one can be considered urban, as it has more than 150 inhabitants/km^2^ [24].

Given the above, this study aims to:

(1) Determine the level of compliance with physical activity recommendations among adolescents according to sex and place of residence.
(2) To determine the perceived support of family and friends for physical activity among adolescents according to sex and place of residence.
(3) To analyse the influence of family and friends’ support on adolescents’ compliance with physical activity recommendations according to sex and place of residence.

## 2. Materials and Methods

### 2.1. Study Design

The study had a cross-sectional design, with descriptive character of the levels of compliance with recommendations of physical activity practice and correlation of compliance with the social support perceived by adolescents regarding their physical activity practice. The ethical principles of the Declaration of Helsinki were followed, and the study was approved by the Ethics Committee of the University of Granada (2966/CEIH/2022). Informed consent was also obtained from the parents and legal guardians of the students, as well as the adolescents’ assent.

### 2.2. Participants

The study population consisted of 3224 adolescents in Soria (Spain) in compulsory secondary education (12–17 years). A non-probabilistic sampling design was used based on the criterion of recruitment by accessibility. To this end, a group of students per year and centre was selected so that all groups from the same centre could complete the questionnaires on the same days. A total of 1089 students from 17 schools participated, excluding those who met any of the exclusion criteria: (1) the day on which the physical activity was asked about was considered unusual by the student, (2) the questionnaire was not answered correctly during the four days of administration, and (3) there were outliers in the data analysis. The final sample consisted of 694 students with an age of 14.06 (±1.27) years. This sample size implies a precision error of 3.3% for a standard deviation of 50 and a confidence level of 95%, according to statistical sampling calculations performed with Epidat 3.1 software. Therefore, the sample can be considered representative of the population under study [25].

Regarding sex, 52.4% of the participants were boys (n = 364) and 47.6% were girls (n = 330). Concerning grade, 24.4% of the students were in grade 1 (n = 169), 25.8% in grade 2 (n = 179), 23.8% in grade 3 (n = 165) and 26.1% in grade 4 (n = 181). Furthermore, 46.25% (n = 321) lived in urban areas and 53.75% (n = 373) in rural areas.

### 2.3. Instruments

The Four-by-One Day Physical Activity Questionnaire was used to measure adolescents’ physical activity levels. This questionnaire was originally developed by Cale [26], but the version validated for Spanish adolescents by Soler et al. [27] was used. It is administered on four days (a winter school day, a winter weekend day, a spring school day and a spring weekend day) and the physical activity levels of each participant are calculated as a four-day average. In addition, one of the two school days must have been spent in physical education and, for the weekend days, one must be answered on Saturday and the other on Sunday. Therefore, the questionnaire has two formats, one for school days and one for weekend days. The questionnaire makes it possible to identify the type of physical activity performed and the time spent on each. In addition, the protocol establishes a categorisation of activities according to their intensity, with moderate–vigorous physical activities being those with an associated energy expenditure of at least 4 METs/hour.

The Parental Support Scale and the Peer Support Scale were used to determine the social support perceived by adolescents in relation to their physical activity practices. These scales were originally developed by Prochaska et al. [28], but the Spanish version by Sanz-Martín [29] was used. Each social support scale consists of five items on a Likert scale of 0–4 (0 = never; 4 = every day) and asks about perceived support during the last week. The five questions of the family members scale are: (1) their family members encourage them to do physical activity, (2) they do physical activity with their family members, (3) their family members provide them with transportation to do physical activity, (4) their family members see them doing physical activity and (5) their family members tell them that their do PA well. The five questions on the friends’ scale are (1) they encourage their friends to be physically active, (2) their friends encourage them to be physically active, (3) they are physically active with their friends, (4) their friends make fun of them for being physically active and (5) their friends tell them that they do PA well.

### 2.4. Procedure

In the present study, the number of days on which adolescents met the recommendations for at least 60 min of moderate-to-vigorous physical activity [2] was calculated. In addition, the dichotomous variable of physical activity compliance was used, which was positive (yes) if the adolescents met the recommendations on the four days the questionnaire was administered. The reliability of the questionnaire was α = 0.832.

The social support scales were administered together with the physical activity questionnaire. Mean scores were calculated for each item on the scales, taking into account ten social support variables. The Cronbach’s alpha reliabilities of the scales ranged from 0.7 to 0.83.

The three instruments used in the study were administered together over four days, were paper-based and followed the physical activity questionnaire protocol, with one researcher responsible for a maximum of six students. The average daily response time was approximately 25 min.

### 2.5. Data Analysis

IBM SPSS 26.0 software (International Business Machines Corporation, Armonk, NY, USA) was used for statistical analysis. Two phases were followed in this analysis: (1) the preparatory and descriptive statistics phase, (2) the creation of predictive models of adherence to physical activity recommendations.

In the first phase, the data matrix was created with the adolescents’ responses to the instrument administered. Descriptive statistics were then calculated for the variables. In addition, Chi-square and Crammer’s V tests were calculated to identify differences in adherence to the recommendations according to sex and municipality of residence, and Student’s *t*-test to do so for social support scores (normal distribution was previously checked with the Kolmogorov-Smirnov test). A statistical significance level of *p* ≤ 0.05 was set. Similarly, Cohen’s d statistic was calculated to determine the size of the effect, which was considered small if it was close to 0.2, moderate if it was around 0.50 and large if it was close to 0.80 [30].

In the second stage, the initial binary logistic regression model was constructed using the following formula: $y=\frac{1}{1+e^{-f(x)}}$; where *f*(*x*) is the physical activity compliance, and in turn *f*(*x*) = *a* + *β*1*variable1 *i*1 + *β*2*variable2 *i*2 + *β*3*variable3 *i*3 + … + *Ꜫi*. Models were identified according to the sex of the adolescents and the type of municipality of residence. The Hosmer–Lemeshow test was then used to test the goodness of fit of the models, with a *p*-value > 0.05 being considered optimal [31]. In addition, the Nagelkerke R^2^ statistic was used to calculate the analysis of variance.

## 3. Results

In Soria, 86.60% (n = 601) of adolescents did not comply with the recommendations for physical activity during the four days of response, compared to 13.40% (n = 93) who complied. Table 1 shows the percentages of compliance according to the sex of the adolescents and their usual place of residence. Regardless of the area of residence, there are significant differences in compliance according to the sex of the students (rural area: x^2^ = 4.192, *p* < 0.05; V = 0.106, *p* < 0.05; urban area: x^2^ = 8.999, *p* < 0.05; V = 0.167, *p* < 0.01). No significant differences in physical activity compliance were found between rural and urban girls. Similarly, no differences were found when comparing these scores for boys.

Table 2 presents the descriptive statistics of the scores for social support from family and friends by students’ sex and place of residence. There are significant differences in the mean scores of boys and girls in rural areas in the items related to family providing transportation (t = 3.878, *p* ≤ 0.001; d = 0.40), friends encouraging (t = 2.254, *p* = 0.025; d = 0.23), friends encouraging (t = 2.796, *p* = 0.005; d = 0.29) and doing physical activities together (t = 4.974, *p* ≤ 0.001; d = 0.50). On the other hand, among students from urban areas, significant differences by sex were found only for doing physical activity with friends (t = 2.109, *p* = 0.036; d = 0.23). There are also significant differences between the scores of boys in rural and urban areas for the item that their friends encourage them to be physically active (t = −3.205, *p* = 0.001; d = 0.18). In contrast, for girls there are differences in the items that their family members encourage them to be physically active (t = 2.433, *p* = 0.016; d = 0.28) and that they provide them with transport (t = 3.406, *p* = 0.001; d = 0.38).

Conditional or stepwise binary logistic regression models were fitted by sex and municipality, with adherence to physical activity recommendations as the dependent variable and all social support variables as variables. Table 3 shows the statistics of these models for girls and boys in rural areas. In both models, only the variable of doing physical activity with friends was included. The formula for the final model was: $y=\frac{1}{1+e^{-f(x)}}$; where for girls, it was *f*(*x*) = −5.3 + 1.131*doing physical activity with friends. The model has a good fit (Hosmer–Lemeshow *p*-value > 0.05) and explains 13% of the adherence to physical activity recommendations (Nagelkerke’s R^2^ = 0.130). The closer the result of the model is to 1, based on the participant’s data, the more likely he/she is to comply with the physical activity recommendations. The model for boys was *f*(*x*) = −4.496 + 0.963*doing physical activity with friends and explains 10% of the variance (Nagelkerke’s R^2^ = 0.100).

Table 4 shows the statistics of the logistic models for girls and boys in urban areas. Based on the initial model formula, for girls *f*(*x*) = −8.557 + 1.141*their family members see them doing physical activity + 1.134*they do physical activity with their friends. The model has a good fit and explains 30.4% of the adherence to physical activity recommendations. In contrast, for boys: *f*(*x*) = −2.525 − 1.730*their friends make fun of them for being physically active + 0.679*their friends tell them they are good at physical activity. In this case, the model explains 16.7% of the variance in physical activity compliance.

## 4. Discussion

The results obtained are discussed below, based on the objectives of the study and the existing scientific evidence.

It was found that more than 86% of adolescents in Soria did not meet the daily recommendations for physical activity. These levels of non-compliance are slightly higher than those found by Guthold et al. [4] for adolescents worldwide (81%) and by Santos-Labrador [32] for young people in Salamanca (82%). Similarly, participants from rural areas achieve a higher percentage of non-compliance with physical activity recommendations than those from urban areas. Therefore, it would be advisable to make general proposals to promote adolescent health, such as increasing physical activity outside school, which is particularly important in rural areas [12].

Regarding the percentages of physical activity adherence according to sex and place of residence, it has been shown that girls living in rural and urban areas have higher levels of inactivity than boys. The existence of higher levels of inactivity in females has been demonstrated in previous studies [4,32]. On the other hand, girls in urban areas and boys in rural areas have higher levels of physical inactivity, although there are no significant differences according to sex and place of residence. Following the same trend, Espejo et al. [33] also showed that rural adolescent boys were less physically active outside school than girls. In contrast, Franco-Arévalo et al. [34] found different results, as they found that the most inactive boys were from urban areas. These differences in physical activity levels by place of residence are also observed globally and may be due to income levels [4], fewer rural areas for sporting activities [12,34] and difficulties in engaging in physical activity with peers, as the average age of the rural population is higher, and the proportion of young people is lower [11].

There are differences in the mean scores for the items on the perceived social support for physical activity scale according to sex and municipality of residence of the young people. Comparing the levels of support for young people in rural areas, there are significant differences for families providing transport, friends encouraging, friends encouraging and doing physical activity together. In contrast, among urban participants, there are significant differences only for doing physical activity with friends, with higher levels for boys. These results in rural areas are similar to those reported by Sanz-Martín [11], who also found significant sex differences in primary school children in rural areas of Zaragoza in the items related to family members providing transport and doing physical activity with friends.

Rural girls perceive less social support from family and friends than urban girls on all items except being teased by friends. Furthermore, these differences are significant for the perceptions that families encourage them to be physically active and that families provide them with means of transport. Comparing these results with those of the study by Sanz-Martín [11], primary school children from remote rural areas of Zaragoza perceive greater encouragement from their families to be physically active, but less encouragement from their families to provide them with means of transport. In this context, the difference in the perception of the share of transport could be conditioned by the location and type of municipality of residence, with a higher perception among students in urban areas, followed by those in rural areas close to urban areas, and finally those in remote rural areas, where they are further away from areas where more physical activity facilities are accumulated [35]. Similarly, the difference in social support between primary and compulsory secondary school students may be because social support tends to decrease with age [36,37].

The social support variable of being physically active with friends is the strongest predictor of adolescents’ adherence to physical activity recommendations. It is the only variable that predicts adherence to physical activity among girls and boys from rural areas. This variable, together with being seen by family members doing physical activity, also predicts 30.4% of adherence to the recommendations. These results confirm the findings of previous studies on the importance of social support from family and friends in the practice of physical activity by young people [38,39], which is considered a mechanism that shapes behaviour [40].

Given the very high levels of physical inactivity among adolescents in Soria, it is considered essential to design and implement health promotion proposals at a general level, but even more so for women and adolescents in rural areas. Based on Bronfenbrenner’s ecological model [16], interventions should be targeted at the different systems and the relationship between their elements. About the exosystem, the mass media could be encouraged to carry out more health promotion campaigns and incorporate new communication systems [41]. Likewise, researchers and specialists in physical education, together with other social actors, should participate in the design of local policies to promote physical activity, as these policies need to be improved, especially in rural areas [42].

At the microsystem level, young people’s families play a special role in promoting physical activity, especially in terms of seeing them do it [11], so work–life balance policies could help to increase social support and physical activity [43]. Another agent of the microsystem that helps to improve physical activity levels is friends, and doing physical activity together is particularly important. In this sense, the “school games” programmes (sports championships with competition by age and school) have had some success in Spain, but they should be updated to increase the number of sports facilities and promotions in schools [44].

At the individual level, proposals to promote physical activity should seek to make it attractive to young people and encourage them to learn about healthy leisure alternatives in areas close to where they live. In this sense, the appropriate use of ICT could help in promotion [45], such as by developing multi-platform mobile applications at the local level to practice physical activity [46].

## 5. Conclusions

The first aim of the study has been met: to determine the level of adherence to physical activity recommendations among adolescents according to sex and place of residence. Most adolescents in the province of Soria do not meet the physical activity recommendations for their age group. Similarly, this trend is maintained regardless of sex and place of residence, with significant differences and higher percentages of non-compliance among girls compared to boys in both rural and urban areas.

The second aim of the study has also been met: to determine the perceived support of family and friends for physical activity among adolescents according to sex and place of residence. The mean scores for most of the items on support from family and friends are higher for boys than for girls in both rural and urban areas. In addition, there are significant differences in favour of boys in rural areas for perceptions of family transport, encouragement from friends, and being active with friends. In urban areas, these significant differences only exist for being active with friends. Similarly, boys in rural areas score significantly higher than boys in urban areas on being encouraged by friends to be physically active. In contrast, girls in urban areas score higher than those in rural areas on the perception that their family members encourage them to be physically active and provide them with transport.

Similarly, the third aim of the study has also been met: to analyse the influence of family and friends’ support on adherence to physical activity recommendations among adolescents according to sex and place of residence. Doing physical activity with friends predicts 13% of rural girls’ and 10% of rural boys’ adherence to physical activity recommendations. In contrast, 30.4% of urban girls’ physical activity compliance is predicted by perceptions of being seen doing physical activity by family members and doing physical activity with friends. In addition, urban boys’ adherence to these recommendations is predicted by scores on being teased by friends and being congratulated for doing physical activity well.

The research carried out has two limitations. One limitation is due to the type of study design, as the cross-sectional design allows us to know the level of lifestyle habits at an exact moment in time, and these may have changed over time. The other limitation derives from the instrument that was used to measure physical activity levels as a subjective instrument: the questionnaire. Although this instrument is not as accurate as others, it has been validated for Spanish adolescents.

Future lines of research can be drawn from the results obtained. It would be advisable to develop proposals to promote physical activity for all adolescents, especially girls and students in rural areas. These proposals could include joint activities between adolescents, friends and family members. Families should also be made aware of the importance of supporting adolescents to be physically active, including the rationale for increasing adolescents’ activity levels and the health benefits. Similarly, longitudinal studies should be carried out to establish the cause–effect relationships of the various factors analysed on physical activity levels, and to include other potential factors such as family income levels or levels of other habits such as screen time and adherence to the Mediterranean diet.

## Figures and Tables

**Table 1:** Descriptive statistics on adherence to physical activity recommendations.

		N (%)	Physical Activity Compliance 4 Days N (%)		Chi-Square	V-Crammer
			Yes	No		
Rural	Girls	186 (26.80)	16 (8.60)	170 (91.40)	4.192 *	0.106 *
	Boys	187 (26.95)	29 (15.51)	158 (84.49)		
	Total	373 (53.75)	45 (12.06)	328 (87.94)		
Urban	Girls	144 (20.75)	12 (8.33)	132 (91.67)	8.999 *	0.167 **
	Boys	177 (25.50)	36 (20.34)	141 (79.66)		
	Total	321 (46.25)	48 (14.95)	273 (85.05)		

Note. *p*-value ≤ 0.05 (*); *p*-value ≤ 0.01 (**).

**Table 2:** Descriptive statistics for perceived social support.

		Fa-S1	Fa-S2	Fa-S3	Fa-S4	Fa-S5	Fr-S1	Fr-S2	Fr-S3	Fr-S4	Fr-S5
Rural	Girls	2.07 (0.85) ^#^	1.63 (0.88)	1.82 (1.11) * ^#^	2.07 (0.88)	2.15 (1.00)	1.93 (0.82) *	1.78 (0.86) *	2.29 (0.85) *	0.28 (0.55)	1.85 (0.88)
	Boys	2.19 (0.89)	1.57 (0.85)	2.28 (1.20) *	2.20 (1.05)	2.31 (1.06)	2.13 (0.90) * ^#^	2.03 (0.86) *	2.71 (0.83) *	0.25 (0.49)	2.01 (0.86)
	Total	2.13 (0.87)	1.60 (0.87)	2.05 (1.18)	2.13 (0.97)	2.23 (1.04)	2.03 (0.87)	1.91 (0.87)	2.50 (0.87)	0.26 (0.52)	1.93 (0.87)
Urban	Girls	2.31 (0.89) ^#^	1.67 (0.87)	2.26 (1.22) ^#^	2.12 (1.01)	2.26 (1.07)	2.07 (0.75)	1.87 (0.85)	2.38 (0.91) *	0.26 (0.66)	1.87 (0.98)
	Boys	2.18 (0.90)	1.65 (0.96)	2.43 (1.13)	2.32 (1.01)	2.38 (1.06)	1.97 (0.89) ^#^	1.74 (0.88)	2.59 (0.89) *	0.32 (0.64)	2.04 (0.93)
	Total	2.24 (0.89)	1.66 (0.92)	2.35 (1.17)	2.23 (1.02)	1.06 (0.06)	2.02 (0.83)	1.80 (0.87)	2.5 (0.90)	0.29 (0.65)	1.96 (0.95)

Note. Values are expressed as means and standard deviations. Family members encourage them to be physically active (Fa-S1); they are physically active with their family members (Fa-S2); their family members provide them with transport to be physically active (Fa-S3); their family members see them as being physically active (Fa-S4); their family members tell them that they do PA well (Fa-S5); they encourage their friends to be physically active (Fr-S1); their friends encourage them to be physically active (Fr-S2); they are physically active with their friends (Fr-S3); their friends make fun of them for being physically active (Fr-S4); their friends tell them that they do PA well (Fr-S5); *p*-value ≤ 0.05 for the difference between boys and girls (*); *p*-value ≤ 0.05 for the difference between rural and urban areas (#).

**Table 3:** Binary logistic regression model statistics for rural students by sex.

Physical Activity Compliance		β	SE	Wald	*p*-Value	R^2^	Chi-Square	H-L
Girls	Fr-S3	1.131	0.370	9.355	0.002	0.130	11.008 (*p* = 0.001)	4.212 (*p* = 0.755)
	Constant	−5.300	1.092	23.539	<0.001			
Boys	Fr-S3	0.963	0.317	9.246	0.002	0.100	11.189 (*p* = 0.001)	7.717 (*p* = 0.462)
	Constant	−4.496	0.997	20.345	<0.001			

Note. Standard error (SE); Hosmer–Lemeshow (H-L); do physical activity with friends (Fr-S3).

**Table 4:** Binary logistic regression model statistics for urban students by sex.

Physical Activity Compliance		β	SE	Wald	*p*-Value	R^2^	Chi-Square	H-L
Girls	Fa-S4	1.141	0.478	5.699	0.017	0.304	20.531 (*p* < 0.001)	6.362 (*p* = 0.607)
	Fr-S3	1.134	0.505	5.047	0.025			
	Constant	−8.557	1.912	20.039	<0.001			
Boys	Fr-S4	−1.730	0.723	5.728	0.017	0.167	19.927 (*p* < 0.001)	3.607 (*p* = 0.824)
	Fr-S5	0.679	0.228	8.883	0.003			
	Constant	−2.525	0.557	20.559	<0.001			

Note. Standard error (SE); Hosmer–Lemeshow (H-L); their family members see them doing physical activity (Fa-S4); they do physical activity with their friends (Fr-S3); their friends make fun of them for doing physical activity (Fr-S4); their friends told them they did physical activity well (Fr-S5).

## Data Availability

The data used to support the findings of the current study are available from the corresponding author upon request.
